# Innate Immune Memory: Time for Adopting a Correct Terminology

**DOI:** 10.3389/fimmu.2018.00799

**Published:** 2018-04-19

**Authors:** Diana Boraschi, Paola Italiani

**Affiliations:** Laboratory of Innate Immunity and Inflammation, Institute of Protein Biochemistry, National Research Council, Napoli, Italy

**Keywords:** innate immunity, innate memory, trained immunity, tolerance, non-specific acquired resistance

## Innate Immune Memory and Resistance to Infections

The concept of innate immune memory, i.e., a change in the reactivity in innate immune cells previously exposed to various stimuli, is well known in plants, invertebrates and also in vertebrates (1). Innate immune memory differs from adaptive memory for many aspects, including the lack of gene rearrangements, the involvement of epigenetic reprogramming, the type of cells involved (innate cells vs. T and B lymphocytes), and the receptors engaged in pathogen/antigen recognition [selective pattern-recognition receptors (PRR) vs. antigen-specific T cell and B cell receptors]. In general, although debatable, innate memory is considered as a non-specific short-lived phenomenon, as opposed to adaptive memory that is long-lived and highly specific.

In plants, innate memory is known as systemic acquired resistance (SAR). In SAR, a localized infectious stimulus recognized by PRR induces systemic resistance to subsequent challenges with the same or unrelated stimuli (2). This “broad spectrum” resistance is the major immune mechanism in plants and is very similar to innate immunity and innate memory in other organisms (3).

In invertebrates, which like plants do not have adaptive immunity, the protective innate immune responses are modulated by previous exposure to infectious stimuli, resulting in an increase of a subsequent response to the same or unrelated challenges, in terms of increased number of phagocytes, upregulation of genes related to enhanced clearance of microbes and/or increased phagocytosis (4–8). Most interestingly, innate memory in invertebrates can last long and pass down from generation to generation, with enhanced reactivity found in the offspring (up to the third generation) after a priming event occurred in parental individuals (9, 10).

In higher vertebrates, the concept of innate memory has been known since the last century, with a wealth of studies describing the effect of “priming,” either *in vivo* or *in vitro*, on the subsequent reactivity of macrophages or monocytes to an unrelated challenge (11–13). We will provide just a couple of examples, including one of our own publications, although these certainly are only a few among many (14, 15). It is interesting to note that many of the initial studies in vertebrates reported a phenomenon not detected in invertebrates, i.e., the priming-induced downregulation of the subsequent responses. Starting from the seminal study of Beeson in 1946 (16), several other studies addressed tolerance after priming with bacterial endotoxin, which results in decreased reactivity of macrophages to subsequent challenges (14). Although endotoxin tolerance was not initially considered as a phenomenon of innate memory, this has become increasingly evident with time (17, 18). Other studies, paralleling the abundant observations in invertebrates and plants, addressed the non-specific enhanced response/resistance to infections that ensues priming (15, 19). The hypothesis is that the induction of tolerance is a compensatory mechanism with the scope of limiting the extension of hyperreaction and tissue damage in the case of repeated or chronic infection, whereas the aim of memory-dependent enhancement is that of improving tissue surveillance and protection in situations of weakness or frailty (20, 21). On the other hand, both memory-induced tolerance and hyperresponse can be involved in the pathological sequelae of innate immunity/inflammation, as seen in sepsis and autoimmunity (22, 23).

Recently, the phenomenon of vertebrate innate memory has experienced a renewed interest (19, 24). Studies showed that, in mouse and human cells, priming with *Candida albicans* or the fungal cell wall component β-glucan non-specifically induce enhanced second responses (25). Also, *in vivo* vaccination with the Gram-positive bacterium Bacillus Calmette–Guérin could induce a more effective host immune response to subsequent challenges, with a concomitant increase in resistance to unrelated infections (26, 27). In agreement with data obtained in invertebrates, studies on the molecular mechanisms underlying the establishment of innate memory show the strong involvement of transcriptional and epigenetic reprogramming, including histone acetylation and methylation and modulation of miRNAs, which can be shaped by environmentally induced metabolic changes (1, 28–33).

It is also important to consider that innate memory can be compartmentalized within the body, with cells retaining memory in some organs but not in others, in agreement with the organ-specific characteristics of innate cells (34). Furthermore, innate memory is not a phenomenon restricted to monocytes and macrophages. Innate lymphoid cells, in particular NK cells, show both non-specific and specific memory features, and mechanistically include both innate and adaptive traits (19, 35). Also, innate memory can be induced at the level of immune stem cells, in bone marrow niches in which non-immune cells likely contribute to inducing stem cell priming (36–38). Eventually, the recent finding that epithelial stem cells retain memory of previous inflammatory challenges by displaying an enhanced wound healing capacity upon skin damage shows that innate memory may not be restricted to immune cells (39).

Thus, innate memory, induced by vaccination or previous exposure to infections or other challenges, may determine the effectiveness of subsequent defensive innate responses in a personalized fashion, dependent on individual history of pathogen/antigen exposure (40). On these grounds, it is promising the finding that LPS-induced tolerance can be reversed, in some individuals, by β-glucan (41). If also the reverse is proven, i.e., if memory-dependent enhancement can be reversed by tolerance-inducing agents, this could open the way to very interesting personalized immunotherapeutic approaches (32).

## Need for a Revised Terminology

The renewed interest raised by the recent developments in the field of innate memory of vertebrates is evident: 1,573 hits for “innate” and “memory” in PubMed since 2011, out of 2,724 since 1946 (by February 1, 2018). Thus, we feel that this is the right time for re-thinking the terminology that we are using for describing the various innate memory phenomena (Table 1).

**Table 1:** Innate immune memory terminology.

Phenomenon	Old terms	Proposed terms
Innate immune memory	Innate memory	Innate immune memory
		Trained innate immunity

Induction of innate memory	Priming	Priming
	Innate immune reprogramming	Innate immune reprogramming
	Pre-conditioning	

Memory-induced decreased responsiveness	Tolerance	Tolerance, trained tolerance (global phenomenon)
		Contraction, decrease (individual effectors)

Memory-induced enhanced responsiveness	Trained immunity	Potentiation, trained potentiation (global phenomenon)
	Non-specific acquired resistance	Enhancement, increase (individual effectors)

Induction of innate memory is the consequence of an innate immune reaction, in which the usual mechanisms of innate immunity are involved, such as recognition of stimuli through receptors specific for pathogen-associated molecular patterns and danger-associated molecular patterns (42, 43). We feel that the term “priming” is still appropriate for describing the phenomenon of memory induction. The term does not define whether we are talking about innate or adaptive immunity, but this can be specified by adding the relevant adjective or by the context. In any case, the term implies the induction of memory (the consequence of an immune response), thereby distinguishing it from the immune response itself. Another excellent way of describing the induction of innate memory is “innate immune reprogramming,” a term that underlines the complex changes behind immune cell reactivity after priming (34, 44).

Regarding the innate memory-induced responses, the terminology is at present partly unclear and would benefit from the use of clear definitions. For instance, the term “trained immunity,” often used for describing enhanced responsiveness, is not precise. Training, i.e., education, is expected to result in a response that is different from the initial one, either higher or lower, either more or less protective. In other words, training can go in both directions.

In the case of decreased responses to a second challenge, as in the case of endotoxin, the term tolerance is widely used. In general, “immunological tolerance” defines the lack of response of lymphocytes to antigens, therefore pertaining to adaptive immune processes. In the case of innate immunity, the term “endotoxin tolerance” is in use since 1946 for describing the lack of subsequent response to endotoxin after a first exposure (12, 16). We must be aware that the term refers to the final outcome of the reaction, i.e., the absence or decrease of a reaction that would otherwise lead to inflammation and eventual tissue damage. Therefore, this mainly applies to *in vivo* outcomes in whole organisms. However, when assessing individual inflammation-related factors such as cytokines, both *in vivo* and *in vitro*, it is evident that tolerance is not a simple decrease in the response to endotoxin. As an example, a study showed that endotoxin tolerance in the mouse is characterized by decrease in TNFα, IL-6, and IFNγ, no change in IL-1β and IL-18, and increase in IL-12, CXCL1, and CCL2 (44). Thus, it must be clear that endotoxin tolerance (similar to immunological tolerance) is the result of a process of general reprogramming of the response, with some effectors being decreased, while others increase or do not change. In this context, the term “tolerance” (or, if we prefer, “trained tolerance”) refers exclusively to the final outcome and cannot be applied to each and every factor and pathway involved in the innate response. When we refer to the individual factors, we should use a different term to describe a decrease, for instance, “contraction” or simply “decrease.”

As already mentioned, in the case of enhanced responses to a second challenge, the memory response is often called “trained immunity,” a term proposed in 2011 by Netea et al. (42). The authors implied that “training” of innate immunity due to previous stimulation (i.e., priming) would result in an enhanced non-specific reaction to subsequent challenges. Thus, the term trained immunity is now used for defining a priming-dependent increase in innate responses. However, we think that trained innate immunity is synonymous of innate memory, not restricted or limited to the enhancement of secondary responses. Thus, while we can certainly continue to use “trained immunity” as an alternative way to define innate memory, it would be important to adopt a different term when referring to a memory-induced enhancement of innate protective responsiveness. The old definition of non-specific acquired resistance is conceptually excellent, but it is a bit cumbersome and lengthy. We therefore wish to propose a one-word term able to convey the concept, and easy to remember and use, for instance, “potentiation” (or, if we prefer, “trained potentiation”). As for tolerance, “potentiation” should refer to the final outcome as it occurs *in vivo* at the level of the entire organism (e.g., increased resistance to infection), because not all the individual innate factors and cells involved undergo an enhancement. In fact, potentiation is the result of a reprogramming of innate reactivities, in which specific mediators can increase, some decrease, or remain unchanged (18, 45, 46). Thus, when referring to the increase of individual effectors we can define it as “enhancement” or “increase.”

In conclusion, it is important to remember trained tolerance and potentiation do not exclusively depend on the priming stimulus (e.g., endotoxin not always induces tolerance) (12, 18, 34, 47, 48). A myriad of environmental factors and other variables affects trained innate memory, including individual history of pathogen/antigen exposure, organ and tissue microenvironment, health and metabolic conditions, gender and age. This would call for a personalized assessment of innate memory responses, before being able to effectively and safely exploit this mechanism for improving resistance to infections in preventive and therapeutic approaches in susceptible populations (49).

## Author Contributions

Both authors have made a substantial, direct, and intellectual contribution to the work and approved it for publication.

## Conflict of Interest Statement

The authors declare the lack of any commercial or financial relationships linked to a potential conflict of interest.
